# An Improved Convolutional Capsule Network for Compound Fault Diagnosis of RV Reducers

**DOI:** 10.3390/s22176442

**Published:** 2022-08-26

**Authors:** Qitong Xu, Chang Liu, Enshan Yang, Mengdi Wang

**Affiliations:** 1 Key Laboratory of Advanced Equipment Intelligent Manufacturing Technology of Yunnan Province, Kunming University of Science & Technology, Kunming 650500, China; 2 Faculty of Mechanical & Electrical Engineering, Kunming University of Science & Technology, Kunming 650500, China

**Keywords:** compound fault diagnosis, convolutional neural network, capsule network, RV reducer

## Abstract

In fault diagnosis research, compound faults are often regarded as an isolated fault mode, while the association between compound faults and single faults is ignored, resulting in the inability to make accurate and effective diagnoses of compound faults in the absence of compound fault training data. In an examination of the rotate vector (RV) reducer, a core component of industrial robots, this paper proposes a compound fault identification method that is based on an improved convolutional capsule network for compound fault diagnosis of RV reducers. First, one-dimensional convolutional neural networks are used as feature learners to deeply mine the feature information of a single fault from a one-dimensional time-domain signal. Then, a capsule network with a two-layer stack structure is designed and a dynamic routing algorithm is used to decouple and identify the single fault characteristics for compound faults to undertake the diagnosis of compound faults of RV reducers. The proposed method is verified on the RV reducer fault simulation experimental bench, the experimental results show that the method can not only diagnose a single fault, but it is also possible to diagnose the compound fault that is composed of two types of single faults through the learning of two types of single faults of the RV reducer when the training data of the compound faults of the RV reducer are missing. At the same time, the proposed method is used for compound fault diagnosis of bearings, and the experimental results confirm its applicability.

## 1. Introduction

Industrial robots are at the core of intelligent manufacturing [1]. As a core component of industrial robots, the health of rotate vector (RV) reducers is an important factor affecting the long-term stable operation of the industrial robots [2,3]. Different from the single fault setting in the laboratory, the different faults are interrelated in an actual operation environment, and compound faults are more common [4] and are the main reason for the failure of the RV reducer [5,6]. The coupling of different types of single faults into a compound fault makes them more difficult to identify [7] and more dangerous than a single fault [8]. Therefore, it is highly significant in the realm of engineering to research compound fault diagnoses of RV reducers.

The RV reducer is composed of a front stage of a planetary gear reducer and a rear stage of a cycloid pinwheel reducer [9]. Due to its complex structure, it is a complicated process to diagnose and identify the damaged parts. Ferrography analysis, acoustic emission analysis, and vibration analysis are the most commonly used methods to monitor the health status of RV reducers [10]. In a ferrography analysis, Peng [11] designed a neural network to classify the wear particles in oil to determine the wear mode of an RV reducer. Although this method determines the wear mode inside the RV reducer, the method is time-consuming and cannot determine the location of a wear failure. In acoustic emission technology, Liang [2] uses a wavelet transform to denoise the acoustic emission signal, and predicts the failure trend of the RV reducer by using a hidden Markov model. An [12] carried out time-frequency feature extraction of acoustic emission signals of an RV reducer at different speeds and working conditions, and used these time-frequency features to qualitatively evaluate the crankshaft wear effect. Yang [13] combined compressed sensing and wavelet energy pooling to extract the fault features of RV acoustic emission signals and implemented a single fault classification of planetary wheel wear and sun wheel wear in an RV reducer. Although acoustic emission technology has achieved good results in early fault diagnoses of RV reducers, the acquisition of acoustic emission signals often requires a very high sampling frequency. A large amount of redundant data has a great impact on data transmission and storage and is not conducive to the long-term condition monitoring of RV reducers. In vibration analysis, since the vibration signal of the machine contains the fault information of the mechanical equipment, vibration analysis is commonly used for fault diagnosis. Many scholars have proposed different fault diagnosis methods and ideas that are based on the characteristics of machine learning algorithms and vibration acceleration signals. Common machine learning algorithms include support vector machines [14,15], artificial neural networks [16], and Bayesian [17]. However, traditional machine learning methods require prior knowledge to manually extract the characteristics of the vibration signals, and the selected feature extraction methods are different for different fault types. Therefore, the diagnostic methods of manually extracting fault features are extremely dependent on experts’ experience and knowledge of fault diagnoses. Moreover, due to the complex structure and working condition identifications of RV reducers, the research on the fault mechanisms of RV reducers is limited, so the results are obtained by methods that are based on a combination of fault mechanisms, and signal processing is relatively limited.

In recent years, deep learning technology, which is widely used in computer vision [18], natural language processing [19], speech recognition [20], and other fields, has been introduced into the fault diagnosis field. End–end intelligent diagnosis modes have become a hot research topic. Methods that are based on deep learning avoid the constraints of the fault mechanism and prior knowledge in feature engineering and achieve good diagnosis effects and performances. Yang [21] reconstructed the one-dimensional vibration signal of an RV reducer into a two-dimensional matrix and used a CNN to mine the two-dimensional matrix for fault features. Peng [2] used dropout to perform random interference on the input signal and fused different features of the input signal for extraction by a multiscale convolution kernel to enhance the feature extraction of networks under strong noise interferences. The identification of planetary wheel wear and cycloid wear of an RV reducer was achieved while under the influence of strong noise. Chen [22] obtained the first four order nonlinear output frequency response functions (NOFRFs) from the vibration acceleration signal of the RV reducer and transformed the spectrum of NOFRFs into a two-dimensional image. CNN was used to extract fault features from a two-dimensional image, and fault classification was carried out for three single faults of a planetary gear frame pitting, cycloid pin wheel pitting and eccentric wheel wear, and two compound faults that were composed of single faults. The above methods have achieved good results in the fault diagnosis of RV reducers, but their fault diagnosis recognition is slightly insufficient. First, faults of several equipment parts are not a mutually exclusive events; that is, the fault of Part A does not mean that Part B will not fail. Second, the relationship between a single fault and a compound fault is ignored, and a compound fault that is composed of a single fault cannot be diagnosed through the learning of a single fault.

In the mechanism of fault generation, a compound fault is not an isolated fault event, and it is closely related to a single fault. A compound fault is also composed of multiple single faults. Yuan [23] showed that a compound fault that is composed of a single fault is the superposition of a single fault vector with different frequencies in the time domain and has multiple single fault feature components in fault characteristics, and the fault characteristics are independent of each other. In 2017, Sabour [24] proposed a capsule network and achieved excellent results on the MNIST overlapping handwritten datasets. Inspired by the identification of overlapping handwritten digit sets by capsule networks and combined with previous research results on the mechanism of compound faults, an intelligent diagnosis method for compound faults of RV reducers that was based on an enhanced convolution capsule network (ECCN) was designed. First, the convolutional neural network is used as the feature extractor, and the one-dimensional single fault vibration signal is used to train the feature extractor. Second, a double stack capsule network is designed as the decoupling classifier to decouple and classify the compound fault features of the RV reducer that were extracted by the feature extractor. Finally, the margin loss function is used to optimize the model. Through an RV reducer fault experiment and the XJTU-SY rolling bearing accelerated life test dataset, the proposed method is verified experimentally, and the effectiveness and superiority of the proposed method are proven. The main research contributions of the proposed method are as follows:

(1) This paper proposes a compound fault diagnosis method for RV reducers that is based on an improved convolutional capsule network. First, the single fault data of the RV reducer are used to train the feature extractor that is composed of a deep convolutional neural network. After training, the feature extractor is used to extract the features of the RV compound faults, and the decoupling classifier that is composed of a double stack capsule network is used to decouple and classify the compound fault features of the RV reducers to implement the learning and diagnosis of compound faults by single faults.
(2) In this paper, the margin loss function is used as the cost loss function of the model to train the model, and the sum of the losses of each type of fault identification is used as the cost loss value. This ensures that the components of the feature set of the extracted fault classes are relatively independent and are not interfered with by other fault features so that the network has an independent fault feature extraction ability.
(3) In this paper, a decoupling classifier that is based on a two-layer stack capsule network is designed, and the proposed features are classified and collected. The squashing function is selected as the normalized activation function of the feature vector, which ensures the independence of the various fault identification and enables the network to have the ability to output multiple tags.
(4) The method in this paper can train the model only with the normal RV reducer and the single fault training dataset when the compound fault data are missing. It can still identify and classify the compound faults that are formed by the combination of single faults and output its single fault component.

This paper’s chapters are arranged as follows. The second chapter briefly introduces the relevant theoretical background knowledge of convolutional neural networks and capsule networks. The third chapter describes the proposed model and the model’s design ideas in detail. In Section 4, the proposed method is verified and analyzed by an RV reducer fault experiment and the XJTU-SY rolling bearing accelerated life test dataset. Finally, conclusions and prospects for future work are given in Section 5.

## 2. Theoretical Background

### 2.1. One-Dimensional Convolutional Neural Network

**Figure 1:**
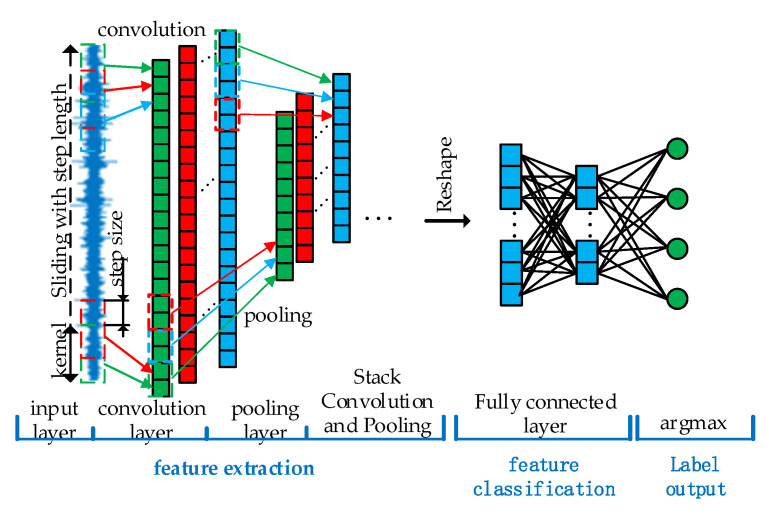
The structure of one-dimensional convolutional neural network.

Unlike traditional neural networks, convolutional neural networks achieve feature extraction of one-dimensional vibration signals by forming a feature extractor from a stack of multiple convolutional and pooling layers. After the fault features are extracted, the features are classified using the full connectivity layer. After the feature classification is completed, the output features are normalized using the softmax function, and the normalized features are labeled using the agmax function. In general, a one-dimensional convolutional neural network [25] contains a total of four key steps: feature extraction, feature classification and label output, and model training, the structure of which is shown in Figure 1.

#### 2.1.1. Feature Extraction

As the core of the feature extractor, the convolution layer mainly includes convolution operation and activation operation. In the convolution operation, the convolution kernel is used as the feature detector, and it is convolved with the input data to obtain a new feature layer. The convolution operation formula is expressed as follows:

(1) $$x_{j}^{l}=\sum_{i}w_{ij}^{l}*x_{i}^{l-1}+b_{j}^{l}$$

$x_{j}^{l}$ is the *j*-th eigenvalue of the *l*-th convolution layer.$\;w_{ij}^{l}$ and $b_{j}^{l}$ are the weights and biases, respectively, and * represents the convolution operation between the convolution kernel and input signal.

So that the network has nonlinear expression capabilities and makes it more conducive to the feature mining of one-dimensional signals, the activation function leakyReLU [26] is used to carry out a nonlinear mapping of the features. This allows the network to mine the negative feature information and have nonlinear feature expression capabilities. An expression is formulated as follows:

(2) yjl=leakyReLU(xjl)=max{0,xjl}+leak∗min(0,xjl)

The value of leak is empirically taken as 0.05.

After the convolution layer, a pooling layer is usually connected. The pooling layer can be regarded as a special convolution operation. An input layer with the size of n×1 is divided into multiple small units of k×1, and the maximum output of each small unit is calculated. A new feature layer y with the size of n/k×1 is formed as $y_{d}^{l}$ to achieve the purpose of a feature reduction of input feature $y_{j}^{l}$ and the elimination of the redundant features to prevent the network from overfitting. The expression is as follows:

(3) $$y_{d}^{l}=\underset{(j-1)k+1\leq t\leq jk}{\max}\left\{y_{t}^{l}\right\}$$

Through layer-by-layer stacking of the convolution layer and pooling layer, the network can learn deeper features that have stronger discrimination and have stronger nonlinear table abilities.

#### 2.1.2. Feature Classification

After the features are extracted, the convolutional neural network uses the fully connected neural network as a feature classifier to classify the proposed features. Before entering the fully connected layer, the learned feature matrix first needs to be expanded and transformed into a one-dimensional feature array. Using the feature array as input to the fully connected layer. The fully-connected layer assigns weights to each feature value in the feature array, thus enabling the transfer of the bottom features to the top features. Its calculation formula is as follows.

(4) Ojl=ReLU(wijlOil−1+bil)

$w_{ij}^{l}$ and $b_{j}^{l}$ are the weights and biases of the fully connected layer. $O_{i}^{l-1}$ is the *i*-th eigenvalue of the output of the previous fully connected or pooled layer.$\;O_{i}^{l}$ is the *j*-th eigenvalue of the output of the fully connected layer. The classification of the bottom features $O_{i}^{l-1}$ into the top features is accomplished through the weight coefficients $w_{ij}^{l}$ and biases $b_{j}^{l}$.

#### 2.1.3. Label Output 

After obtaining the output features, the softmax function [27] is used to normalize the output features $O_{j}^{l}$. The mathematical expression of the Softmax function is as follows:

(5) $${\hat{O}}_{j}=\mathrm{soft}\max(O_{j}^{l})=\frac{\exp(O_{j}^{l})}{\sum_{j=1}^{C}\exp(O_{j}^{l})}$$

$\hat{O_{j}}$ is the feature obtained after softmax normalization. The argmax function [5] is used to find the maximum $\hat{O_{j}}$ for label output, so as to clarify the type of fault, and the label output is calculated as follows:

(6) $$\mathrm{label}=\arg\max({\hat{O}}_{j})$$

#### 2.1.4. Model Training

After building the convolutional neural network model, the network model needs to be trained. The weight parameters in the neural network are optimized to achieve the goal of fault classification. The convolutional neural network is trained with cross entropy as the cost loss function of the model. The optimal combination of parameters is found by finding the minimum loss value of the model. Suppose that given a training set ${\left\{x_{i},y_{i}\right\}}_{i=1}^{M},$ *M* is the number of samples, sample $x_{i}$ corresponds to label $y_{i}\in \left\{1,2,3..,C\right\}$. *C* is the number of categories. The cross-entropy loss [28] is calculated as follows:

(7) $$J(w,b)=-\frac{1}{M}\left[\sum_{m=1}^{M}\sum_{C=1}^{C}1\left\{y_{m}=c\right\}\log({\hat{O}}_{j})\right]$$

1{∗} is the indicator function returns, a class classification correct return value of 1, classification error return value of 0. 

### 2.2. Capsule Network and Dynamic Routing Algorithm

**Figure 2:**
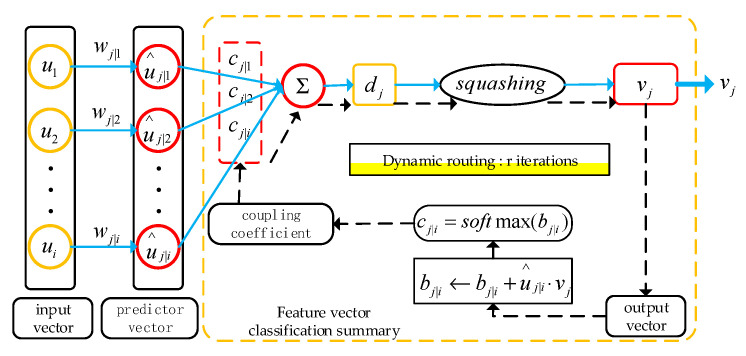
Dynamic routing algorithm.

The core idea of the capsule network [24] is to transform the traditional scalar neurons into vector neurons and take the vector as the input and output of the network to reduce the loss of the feature information in the transmission process to improve the recognition ability of the network. The capsule network consists of two layers of capsule layers. The feature vector is transmitted between the underlying neurons and the upper neurons by a dynamic routing algorithm. The principle of the dynamic routing algorithm is shown in Figure 2. The entire whole operation process can be divided into four stages:

In the first stage, there is an expansion of the feature matrix that is obtained by the feature extractor to obtain the feature vector $u_{i}$**.** The feature vector $u_{i}$ is multiplied by the weight $w_{j|i}$ to obtain the prediction vector ${\hat{u}}_{j|i}$. $w_{j|i}$ encodes the important space and other relations between the underlying eigenvector $u_{i}$ and the high-level vector $v_{j}$, and its expression is as follows:

(8) $${\hat{u}}_{j|i}{=w}_{j|i}*u_{i}$$

In the second stage, the output vector $d_{j}$ is obtained by a weighted summation of the prediction vector ${\hat{u}}_{j|i}$, and its expression is shown in Equation (10), where $k_{l}$ is the number of input feature vectors, and $c_{j|i}$ is the coupling coefficient. The intent is to assign a coupling coefficient to the underlying feature vector so that the underlying feature vector can be more reasonably classified into the matching upper feature vector. In the formula, the sum of all the coupling coefficients $c_{j|i}$ is 1, and the value of $c_{j|i}$ is obtained by updating the value of $b_{j|i}$ through dynamic routing. The formula is as follows:

(9) $$c_{j|i}{=\mathrm{softmax}(b}_{j|i}{)=\exp(b}_{j|i})/\sum_{n=1}^{i}\exp{(b}_{j|n})$$

(10) $$d_{j}=\sum_{i=1}^{K_{l}}c_{j|i}{\hat{u}}_{j|i}$$

In the third stage, by using the squashing function, a nonlinear mapping is carried out on the output vector $\;d_{j}$, and the modulus of its output vector is normalized to 0~1, to obtain the output vector $v_{j}$:

(11) $$v_{j}=\frac{||d_{j}|{|}^{2}}{1+||d_{j}|{|}^{2}}\frac{d_{j}}{\left||d_{j}|\right|}$$

In the fourth stage, which is dynamic routing, the similarity between the predicted feature vector ${\hat{u}}_{j|i}$ and the output feature vector $v_{j}$ is calculated by the inner product to optimize the update $b_{j|i}$, as shown in Formula (12).

(12) $$b_{j|i}\leftarrow b_{j|i}+{\hat{u}}_{j|i}\cdot v_{j}$$

If the similarity between the predicted feature vector ${\hat{u}}_{j|i}$ and the output feature vector $d_{j}$ is higher, the value of $b_{j|i}$ is larger, and then the coupling coefficient $c_{j|i}$ corresponding to the predicted feature vector ${\hat{u}}_{j|i}$ is increased by Formula (9). If the similarity is lower, the value of $b_{j|i}$ is reduced. Through continuous iterative optimization, the optimal coupling coefficient $c_{j|i}$ is obtained so that the bottom feature vector can be better classified and clustered into the upper similar feature vector, and the final output feature vector $v_{j}$ is obtained. The modal length of the output feature vector $v_{j}$ is the probability of the existence of the jth class. $p_{j}=\|v_{j}\|=\sqrt{v_{j}{}^{2}}$

## 3. The Proposed Fault Diagnosis Method

In recent years, convolutional neural networks have achieved many good results in the field of fault diagnosis with their powerful feature extraction ability. As a feature classifier for convolutional neural networks, fully connected neural networks have a powerful nonlinear fitting capability and can formulate the classification model in detail on the problem of fault feature classification. However, due to the large number of parameters of the fully connected neural network, it is easy to lead to overfitting of the network model, which lacks robustness in fault identification and cannot effectively identify unknown faults with large variability. Therefore, the traditional convolutional neural network cannot effectively identify compound faults in the absence of compound fault training data. As shown in the literature [2,22], although the convolutional neural network has learned two kinds of single faults of cycloid pin wheel pitting and planetary wheel pitting of RV reducer, it is unable to identify the compound fault that is composed of cycloid pin wheel pitting and planetary wheel pitting by learning two kinds of single faults. At the same time, due to the limitation of Equations (5) and (6), the classifier of the traditional convolutional neural network can only label the largest fault feature, and cannot guarantee the independence of fault identification, so it cannot completely identify the single fault component in the composite fault signal. 

**Figure 3:**
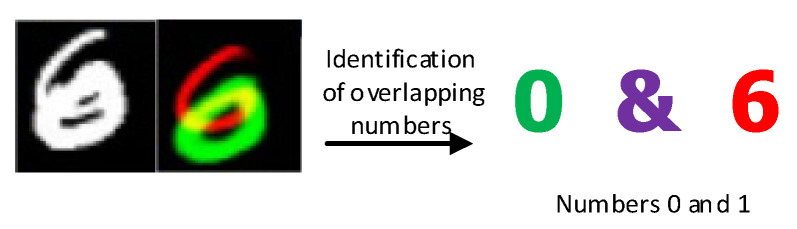
Capsule network identifies overlapping digits.

**Figure 4:**
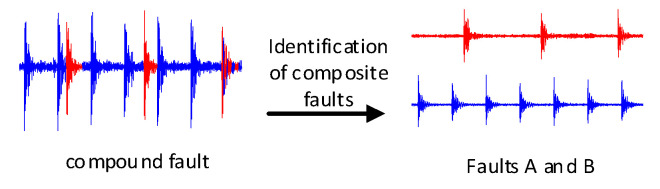
Identify faults A and B.

In 2017 Sabour Proposed the Capsule Network. The capsule network has a strong ability for feature classification and identification, which can identify the composition of overlapping numbers through single-digit learning, and conduct multi-label output, resulting in the effect shown in Figure 3. This is an important inspiration for the identification of compound faults. Can the network diagnose a compound fault consisting of a combination of two single faults of the RV reducer through the learning of a single fault of the RV reducer, and when a compound fault occurs the network outputs the single fault component inside the compound fault to achieve the effect as shown in Figure 4. Inspired by the literature [8], and through an in-depth study of Compound fault mechanisms. This paper improves the traditional convolutional neural network, combines the powerful feature extraction ability of the convolutional neural network with the excellent feature classification ability of the capsule network, and replaces the fully connected layer of the traditional convolutional neural network with the capsule layer to further improve the feature classification ability of the convolutional neural network enables the network model to identify the composite faults composed of single faults through the learning of single faults when the composite fault data is lacking. Solve the problem that the traditional convolutional neural network cannot effectively identify the composite fault due to the lack of composite fault training data.

### 3.1. Model Structure

**Figure 5:**
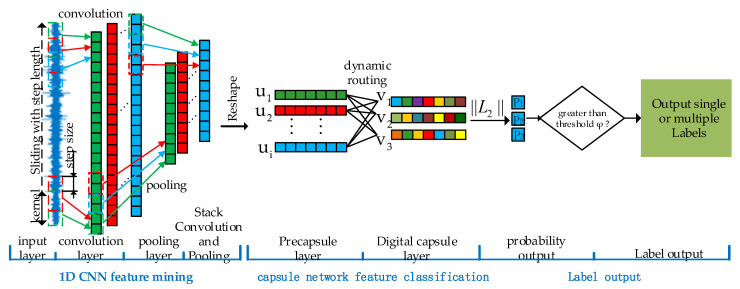
ECCN network structure diagram.

In the network structure, ECCN uses a convolutional neural network as a feature extractor, which is responsible for mining more useful information from the original signal, while avoiding an overreliance on prior knowledge and the complex manual selection of the traditional feature engineering methods. As a compound fault decoupling device, the capsule network can match and identify compound faults based on the characteristic information of a single fault. The fault diagnosis of the RV reducer is implemented by combining a convolutional neural network with a capsule network. The proposed method includes four steps, feature extraction, feature classification, label output, and model training. The network structure is shown in Figure 5.

The entire ECCN model consists of two convolution layers and two capsule layers. In the feature extraction layer that is composed of convolutional neural networks, to obtain more feature information, a larger convolution kernel is used to increase the receptive field of the network. This can reduce the influence of data noise in vibrations to a certain extent to improve the anti-noise ability of the model. The detailed parameters of the model are shown in Section 3.2. 

In feature classification, traditional convolutional neural networks use fully connected neural networks to categorize and subset features, while the feature matrix is transformed into a one-dimensional feature array before entering the fully connected layer, and the neurons are passed between each other by scalar operations, resulting in the loss of many feature-to-feature vector information. To address this problem, this paper designs a two-layer stacked capsule network as a classifier for compound fault features. The feature vector is used as the carrier of feature classification, and the dynamic routing algorithm is used to calculate the similarity between the feature vectors to achieve the feature classification. Compared with a simple scalar operation, the vector operation of the capsule network can use more detailed information for fault recognition, which is the key to implementing the matching and recognition of compound faults through single fault learning.

On the output of the results, the softmax classifier that is widely used in convolutional neural networks can only output single-label fault features, which cannot guarantee the independence of the output of each feature. To address this problem, this paper uses the squashing function to independently normalize the output vector to ensure the mutual independence of feature recognition among the fault classes without interfering with each other. At the same time, the output feature vector is labeled by norm, so that the network has multilabel output abilities.

After iterating the output feature vector v through the dynamic routing algorithm, the modal length of each output vector v is calculated to obtain the final predicted probability value $p_{pred}=\left[p_{1},p_{2},\cdots ,p_{c}\right]$. Each $p_{i}$ in $p_{pred}$ represents the probability that the input sample belongs to class *i*, and the closer the value of $p_{i}$ is to 1, the greater the probability that the sample belongs to class *i*. 

Each type of fault has a prediction probability value, and to limit the number of output labels, a confidence threshold φ is set to limit the number of output prediction labels. If $p_{i}$ is greater than the selected confidence threshold *φ*, it means that the *i*-th class exists and the *i*-th class labels are output. In contrast, the opposite result means that the class does not exist and does not output the label. To obtain reliable classification results, the maximum likelihood estimation method is usually used to give a large confidence threshold *φ*. However, a larger confidence threshold means less prediction and a higher error rate. Therefore, this paper designs an adaptive confidence threshold according to the independence of each fault occurrence and defines the average probability of all the fault classes as the confidence threshold. The formula is described as follows:

(13) $$\varphi =average(p_{pred})=\frac{1}{C}\sum_{i=1}^{C}p_{i}$$

**Figure 6:**
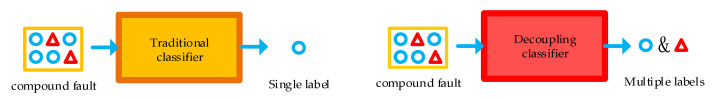
The biggest difference between traditional classifier and decoupling classifier.

To further illustrate the superiority of the proposed method, this paper uses Figure 6 to illustrate the fault characteristics of the RV reducer. As shown in Figure 6, assuming that the fault characteristics of the planetary wheel wear and solar wheel wear of the RV reducer are represented by a circle and triangle, respectively, the compound fault of the RV reducer includes two kinds of fault characteristics of planetary wheel wear and solar wheel wear. Traditional fully-connected layer classifiers are trained and identified based on one data class, and, therefore, cannot identify the difference between compound faults and single faults. Therefore, it can only identify and output the most feature or the most obvious fault type in the compound fault; that is, it cannot output the multilabel output of the compound fault. The proposed method in this paper consists of a convolutional neural network and capsule network, and the obtained network can effectively match and identify the characteristics of a single fault from the compound fault and perform multilabel outputs according to the identified fault to achieve the goal of multilabel outputs of compound faults.

### 3.2. Model Training

ECCN uses the cost function of the margin loss function [29] as the cost function of the multilabel prediction. Compared with the cross entropy cost loss function, the margin loss function cost function that is based on Euclidean distance can directly measure the similarity between the categories. This loss function expands the difference between classes, effectively reduces the variation within the class, and thus improves the diagnostic accuracy of the network. The mathematical expression of the margin loss function is as follows:

(14) $$J=\sum_{c=1}^{C}L_{c}=\sum_{c=1}^{C}\left\{T_{c}\max{\left(0,m^{+}-\left|\right|v_{c}||\right)}^{2}+\lambda (1-T_{c})\max{\left(0,||v_{c}||-m^{-}\right)}^{2}\right\}$$

$T_{c}$ is an indicator function, and if $T_{c}$ is 1, category c exists, and if it is equal to 0, category c does not exist. $||v_{c}||\;$ indicates the probability of identifying the fault classes. $m^{+}$ and $m^{-}$ represent the upper and lower bounds of $\left||v_{c}|\right|$, respectively, and *λ* represents the regularization parameter reduction of a class loss of an object. In this paper, $m^{+}$= 0.9, $m^{-}$ = 2, and *λ* = 3, meaning that when a class of objects exists, the $\left||v_{c}|\right|$. value should not be less than 0.9, and when a class of objects does not exist, the $\left||v_{c}|\right|$ value should not exceed 0.1.

### 3.3. Fault Diagnosis Process

**Figure 7:**
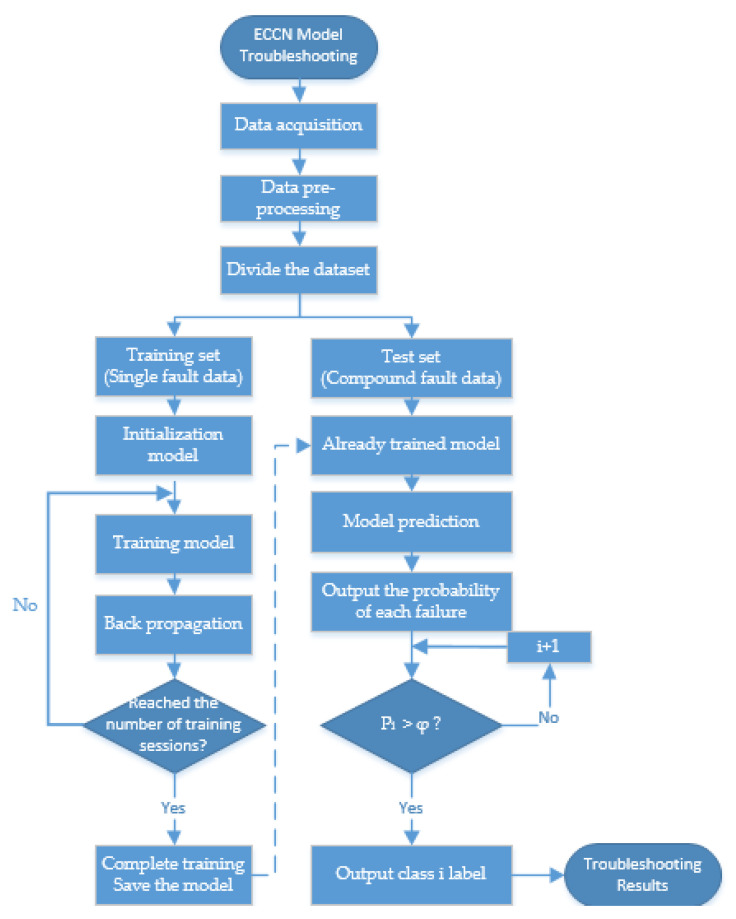
Fault diagnosis flowchart of ECCN model.

In this paper, a deep learning network that can be used for compound fault diagnosis of RV reducer faults is constructed by improving the convolutional neural network. Its diagnostic flow chart is shown in Figure 7. The specific steps of ECCN fault diagnosis are as follows: (1) data acquisition; (2) data preprocessing; (3) dividing the dataset into a training sample set and a test sample set, in which the training sample only contains single fault data and does not contain compound fault data; (4) design the model structure and initialize the parameters; (5) use the training set to train the model and optimize the model parameters by calculating the loss function and backpropagation; (6) test the model with a test dataset containing composite failure data; (7) output the probability of occurrence of each fault; (8) determine whether the probability value of the *i*-th type is greater than the threshold *φ*, if it is greater than the *i*-th type of fault exists; and (9) output fault label, get fault diagnosis results.

## 4. Experimental Verification

### 4.1. Experimental Apparatus and Data Description

**Figure 8:**
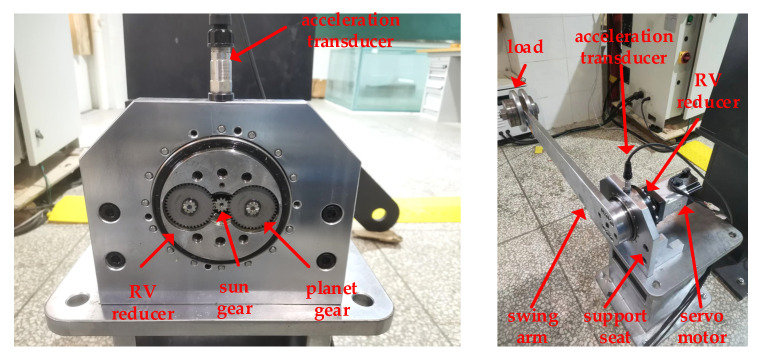
RV reducer fault simulation experimental bench.

To verify the effectiveness of the method that is proposed in this paper, a test was first carried out on the RV reducer fault simulation experimental bench. The test bench comprises of five parts, a load, swing arm, support seat, servo motor, and RV reducer, as shown in Figure 8. The servo motor drives the RV reducer to drive the swing arm to perform a reciprocating rotation. In order to be closer to industrial practice, the RV reducer drives the swing arm to do reciprocating motion in the working condition design. The operation angle range is 0~90°, and the maximum rotation speed is 100°/s. From the initial 0° to the limit position 90°, the swing arm goes through three operating states acceleration, steady speed, and deceleration. From the limit 90° to the initial position 0°, the swing arm also goes through three operating states acceleration, steady speed, and deceleration.

**Figure 9:**
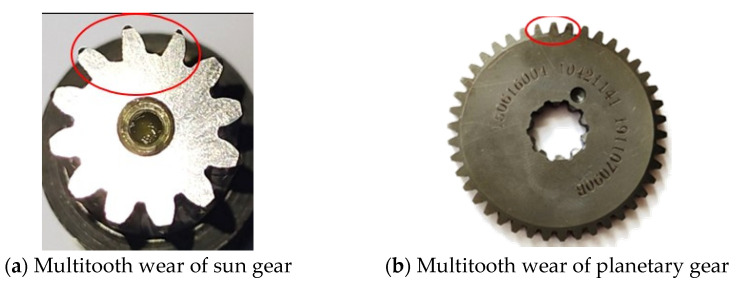
RV reducer fault pictures.

The planetary wheel and solar wheel are the two core parts of the RV reducer. Due to long-term operation in heavy loads and time-varying working conditions, the contact area of the two gears is prone to damage [6]. Therefore, this paper takes the wear fault of the planetary wheel and sun wheel as the research object. Single fault processing is carried out on the sun wheel and planetary wheel of the RV reducer by using WEDM technology. The processing sizes are 0.5 mm, 0.3 mm, and 0.1 mm, which are used to simulate faults with different wear degrees. The fault pictures are shown in Figure 9.

**Figure 10:**
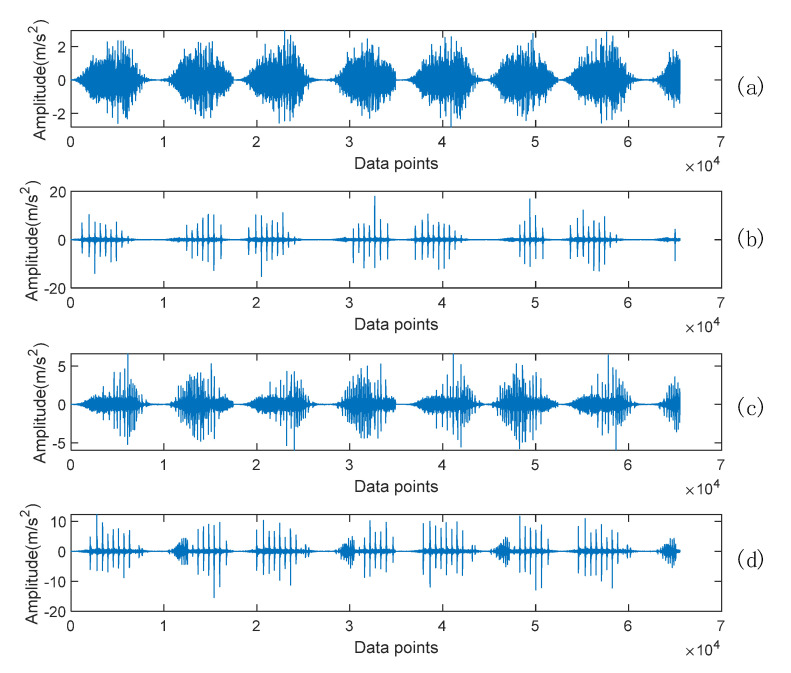
Vibration signal of the RV reducer in four healthy conditions: (**a**) normal condition, (**b**) multitooth wear of planetary gear, (**c**) multitooth wear of the sun gear, and (**d**) compound fault.

This RV reducer has four states: normal, multitooth wear of the sun gear, multitooth wear of planetary gear, and compound fault (multitooth wear of the planetary gear and multitooth wear of the sun gear). The acquisition card is a 9234 acquisition card, the sensor is an ICP acceleration sensor, the sensor number is IMI_603C01, the sensitivity is 100 mV/g, and the acceleration sensor is calibrated using the US PCB handheld acceleration sensor calibrator 394C06 before data acquisition. The sampling time is 26 s and the sampling frequency is 6400 Hz. The time domain diagram of the vibration signal of the RV reducer under four working conditions is shown in Figure 10.

The RV reducer drives the swing arm to make a reciprocating motion. A reciprocating motion takes 2.7 S. At the sampling frequency of 6400 Hz, the RV reducer contains 17,280 data points in one operation cycle. To ensure the speed and recognition efficiency of the network, the number of data points for a set of training data should be $2^{n}$ and contain at least one run cycle, so the data length for each set of training data should be set to 32,768.

**Figure 11:**
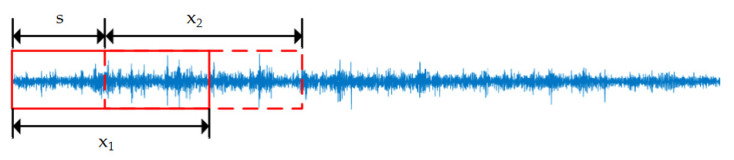
The overlap slicing method.

After the training data length is determined, the data enhancement of the 1D vibration signal of the RV reducer is performed using the overlap slicing method. Data enhancement can increase the training data and improve the model’s generalization ability. In data enhancement, the equal data length window is used to divide the data of one-dimensional vibration signal, and more data samples are obtained by moving the window. The window moves one step s forward to get a data sample $x_{i}$ until sufficient data samples are obtained. In this experiment, the data length of the window is 32,768, and the step size is 64. The detailed visualization of the overlapping slice method is shown in Figure 11:

After obtaining sufficient sample data, the sample data were normalized by z-score. The standardized data were used as the input data for the ECCN network, and the z-score standardization formula was:

(15) $$Y_{i}=\frac{x_{i}-\bar{x}}{\sigma }$$

In the formula: $Y_{i}$ is the standardized data, $x_{i}$ is the original data $\bar{x}\;$ is the original data mean, and σ is the original data variance.

After data preprocessing, TensorFlow generates the training and test sets of the network. Due to the complex operation state and the load moving with the swing arm during the operation, the force condition of the RV reducer is constantly changing. This leads to the RV reducer running in a non-stationary state, the difference in data is significant, and the small amount of training data cannot effectively identify the state of the RV reducer. After several tests, when the number of training sets reached 1000 sets, a better result was achieved for the fault state identification of the RV reducer. 

The fault types include normal, planetary gear multi-tooth wear, sun gear multi-tooth wear, and compound fault. Each type of fault has 2000 samples except for the compound fault. Each sample contains 32,768 data points. To verify the model’s generalization ability, the training set and the test set are divided according to the ratio of 1: 1. The composition of the training and test sets is shown in Table 1. 

It is worth noting that the compound fault data are not involved in the model training during the whole experiment, and the whole training set only includes the vibration data of the RV reducer in three states: normal, planetary wheel wear, and sun wheel wear. After the model training, the compound fault data sample will be used to test the decoupling classification performance of the ECCN model.

### 4.2. ECCN Model Parameters

As described in the second part, the model is divided into four key steps, as follows:

(1) The first step of feature extraction: The design of a dimensional convolutional neural network is to learn and extract the features with depth discrimination and sensitivity from the original vibration signal. In this experiment, two convolution pooling layers are designed. Convolution layer 1 uses the 150 × 1 wide convolution kernel to extract the feature of the signal to reduce the influence of noise [30]. Convolution layer 2 uses a large number of 8 × 1 narrow convolutions to mine the underlying features to extract the deep features of the signal. At the same time, to reduce the training parameters of the model and improve the training speed, a pooling layer is added after each convolution layer for feature reduction;
(2) The second step is feature classification: The capsule networks with sizes of 8 × 12 and 3 × 16 are stacked to form a decoupling classifier to classify and collect the feature vectors that are extracted by the feature extractor;
(3) The third step is label output: the output layer solves the *L*_2_ norm of the output feature vector to obtain the probability of various faults;
(4) The fourth step is model training: the margin loss function is used as the cost loss function to train the model. The maximum number of training iterations is 20, and the batch size is 64. The Adam optimizer is used to train the model.

The proposed methods were run under the Spyder platform of Anaconda software, and the deep learning frameworks were TensorFlow 1.14.0 and Keras 2.2.4. The computer hardware configuration was Intel Core i7-6700 CPU @ 3.4 GHz dual-core CPU with 32 GB memory. The entire model uses the kears toolbox to build the network model. The detailed parameters of the model structure are shown in Table 2.

### 4.3. Experimental Results and Analysis

To verify the effectiveness of the proposed ECCN model in the compound fault diagnosis of RV reducers, this paper selects a CNN for experimental comparison. In terms of model parameters, except for the loss function and classifier, the other parameters of the CNN model are consistent with those of the ECCN. In addition, this paper also selects the existing compound fault diagnosis methods DDCN [8] and DECN [31] to verify the performance of the above model in the fault diagnosis of RV reducers. In model training, all the models only use single fault training samples, including normal, multitooth wear of the sun gear, and multitooth wear of the planetary gear to train the model. CNN, DCCN, DECN, and ECCN are tested by using test samples, including single fault and compound fault. Each model is tested ten times, and the average value of the ten experiments is taken as the model’s accuracy. The diagnostic results are shown in Table 3. The average accuracy of ECCN is 98.50%, and the average accuracy of the other three models is 70.25%, 71.5%, and 92.75%, respectively. In terms of the average accuracy, ECCN is 5.75% higher than the DDCN with the best effect among the three comparison models, and ECNN is 7% and 5% higher than CNN and DDCN in the single fault diagnosis of planetary wheel wear and solar wheel wear, respectively. In compound fault diagnosis, ECCN has been greatly improved compared with other methods, and the DCNN with the best effect in the comparison models increased the accuracy of the compound fault identification by 5%. Due to the limitation of the softmax function, CNN cannot output multiple labels for compound faults, so it is not compared.

**Figure 12:**
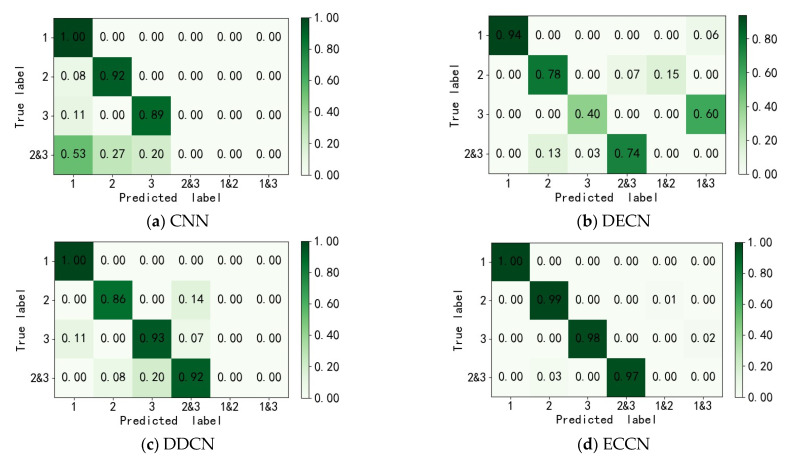
RV reducer data classification confusion matrix (**a**) CNN; (**b**) DECN; (**c**) DDCN; (**d**) ECCN.

The classification confusion matrix includes classification accuracy and misclassification error, which are important metrics for testing the classification results. In Figure 10, the ordinate of the confusion matrix represents the real label of the sample, the abscissa represents the prediction label of the model, and labels 1, 2, and 3 represent the normal, planetary wheel wear, and solar wheel wear of the RV reducer, respectively. Labels 2&3 represent the compound fault label that is composed of the planetary wheel wear and solar wheel wear. Other labels are similar in turn. The color column on the right side represents the corresponding relationship between the value and the color.

(a), (b), (c) and (d) in Figure 10 are the classification confusion matrices of CNN, DECN, DCNN, and the proposed method ECNN, respectively. In Figure 12a, the accuracy of the traditional CNN in single fault identification is above 89%, and the identification effect is good. However, in the compound fault identification, 53% of the compound fault data are identified as normal data, 27% of the compound fault is identified as planetary wheel fault, and 20% of the compound fault is identified as solar wheel fault, which cannot effectively identify the compound fault data.

In Figure 12b,c, DECN and DDCN have a good improvement in the effect of compound fault identification compared with CNN. The accuracy rates of DECN and DDCN in planetary wheel wear fault identification are 78% and 86%, respectively, which are lower than those of the CNN model (92%). In the identification of the solar wheel wear fault data, DECN outputs 60% of solar wheel wear faults with multiple labels, and the errors are identified as normal and solar wheel wear. As shown in Figure 12d, the proposed ECCN method not only achieves 99% and 98% recognition rates of single faults such as planetary wheel wear and solar wheel wear but also achieves 97% recognition rates of compound faults without the participation of compound fault data in training. It completely exceeds CNN in the recognition of compound faults and increases by 5% compared with the better DDCN in the comparison model. It is proven that the proposed method can not only diagnose a single fault, but it is also possible to diagnose the compound fault that is composed of two types of single faults through the learning of two types of single faults of the RV reducer when the training data of the compound faults of the RV reducer is missing.

**Figure 13:**
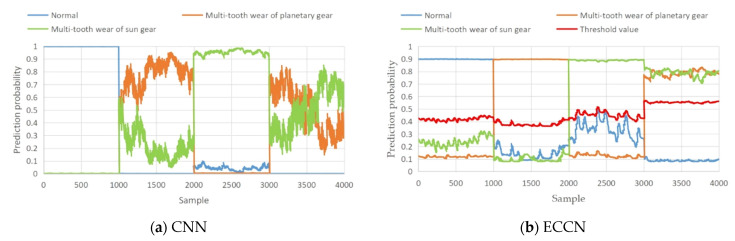
The predicted probability of the model output (**a**) CNN; (**b**) ECCN.

To further illustrate the multilabel output capability of ECCN for compound faults, the CNN and ECCN models are taken as examples for visual analysis. The specific steps are to extract once from the ten experiments and compare the predicted probability values on the visual test dataset, as shown in Figure 11. The abscissa in Figure 11 represents the sample points. The 0–1000 group data belong to the normal test data sample. The 1001–2000 group data belong to the planetary gear fault sample. The 2001–3000 group data belong to the solar gear fault sample. The 3001–4000 group data belong to the compound fault sample, and the ordinate represents the prediction probability value of the model for various types of faults. The red line in Figure 13b is the threshold that is described in the label output of Section 2. When the predicted probability value of a certain type of output of the model exceeds the threshold, it indicates that this type of fault exists.

As shown in Figure 11, it can be seen from Figure 11a that the CNN has good recognition of the normal samples of the 0~1000 group and the planetary gear fault samples of the 1001–2000 group. The normal and planetary gear fault labels are the output by the softmax function, which is consistent with the actual label. However, on the 3001–4000 group of compound fault samples, part of the compound fault samples that were identified by CNN are identified as planetary wheel wear and part of the compound fault samples are identified as sun wheel wear, which cannot produce multilabel output, that is, it adheres to the limitation that is mentioned in Chapter 1. From Figure 13b, it can be seen that the predicted probability values of two faults in ECCN exceed the selected threshold on the Group 3001–4000 compound fault data. According to Section 3 (Formula (13)), two probability values exceed the threshold and the model outputs two labels; namely, the sun wheel wear fault and the planetary wheel wear fault, which are consistent with the actual fault labels. It is proven that ECCN not only identifies the compound fault that is composed of planetary wheel wear and solar wheel wear but also outputs the label number of its single fault component so that the fault diagnosis of the RV reducer by the network model is closer to industrial practice.

The advantages of the ECCN method in complex fault diagnosis are analyzed. The main advantages are as follows:

(1) On the feature normalization and label output, the traditional CNN selects the softmax function (Formula (5)) to normalize the output features, resulting in the probability sum of all the fault categories being 1. The occurrence of the solar and planetary gear faults is forced to be regarded as a mutually exclusive event, and the fault features cannot be output independently.
(2) In addition, in terms of label output, the traditional CNN uses the argmax function (Formula (6)) to index the maximum value of the output feature, so that the network can only output the fault feature with the strongest feature. Therefore, as shown in Figure 4 and Figure 13a, the CNN classifier can only output a single fault label with the largest probability in the compound fault sample, and a fault label with a weak fault will not be able to output. The proposed ECCN uses the squashing activation function (Formula (11)) to independently normalize the fault characteristics and uses the L2 norm to independently output the occurrence probability of each fault, ensuring the independence of each fault identification. Therefore, the ECCN can independently identify and output the fault characteristics of planetary wheel wear and solar wheel wear in compound faults and implement the multi-label output of compound faults, as shown in Figure 13b.
(3) In terms of the training loss function, the traditional CNN uses the binary classification cross entropy-loss function (Formula (7)) to train the model. When a certain type of fault exists, the loss value of other types of faults is zero, resulting in a strong mutual exclusion of the extracted features of the trained model. ECCN uses the margin loss function (Formula (14)) to train each fault class, which ensures that the fault features that are extracted from the various faults are relatively independent and avoids the problem of being unable to identify compound fault information.

### 4.4. Added Experiments

**Figure 14:**
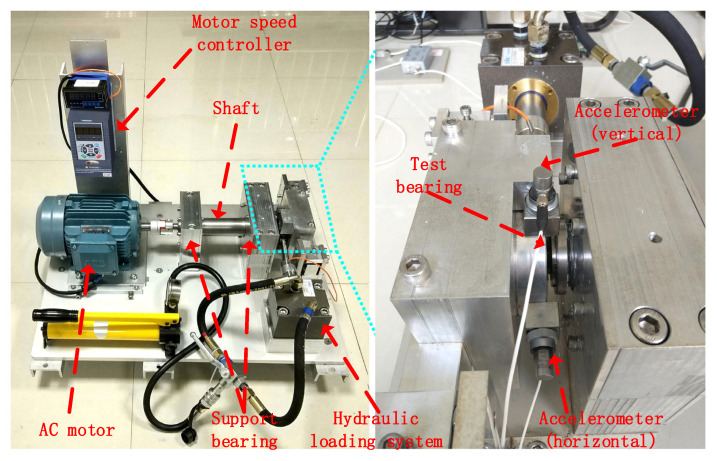
XJTU-SY bearing accelerated life test bench.

To verify the universality of the proposed method, the XJTU-SY rolling bearing accelerated life test dataset is used to verify the proposed method. The dataset of the XJTU-SY rolling bearing accelerated life test is from Xi’an Jiaotong University. The experimental platform is shown in Figure 14 below. The experimental platform is mainly composed of an AC motor, motor speed controller, shaft, support bearing, hydraulic loading system, and test bearing. The detailed parameters of the test bench and data introduction are in Reference [32]. 

**Figure 15:**
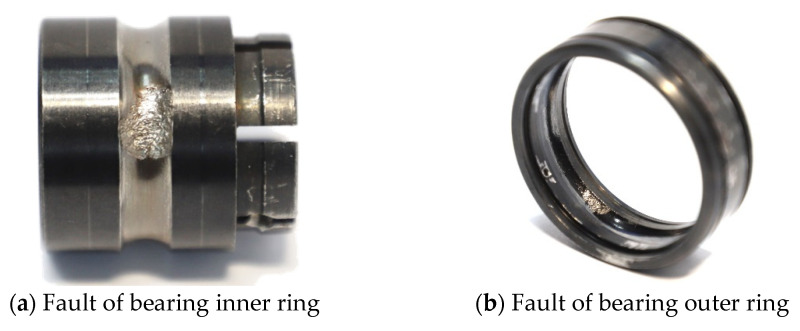
Pictures of bearing failure: (**a**) fault of the bearing inner ring; (**b**) fault of the bearing outer ring.

The accelerated life test dataset of the XJTU-SY rolling bearing has 15 sets of bearing life-cycle data. The failure modes of bearing1_1, bearing2_1, and bearing1_5 are the outer ring fault, inner ring fault, and inner and outer ring compound fault, respectively. In this experiment, the last set of data from Bearing1_1, Bearing2_1, and Bearing1_5 full-life data are selected as the fault data. The real fault data are used to test the effectiveness of the ECCN model on compound fault diagnosis. The failure picture is shown in Figure 15, and the data of the experiment are described in Table 4. There are four state data: normal, inner ring fault, outer-ring fault, and inner-ring and outer-ring compound fault. In addition to the inner and outer ring compound fault, each state generates 200 training data and 200 test data, the sample length is 4096, and the sample partition rule is the same as Section 3.1.

It is worth noting that the 200 compound fault data are only used for model testing and are not involved in model training. The normal data in Table 4 are taken from the first data in the Bearing1_5 life-cycle data. At the time of the experiment, the bearing has not been damaged in the normal state at the beginning of the experiment, so it is selected as the normal sample data.

**Figure 16:**
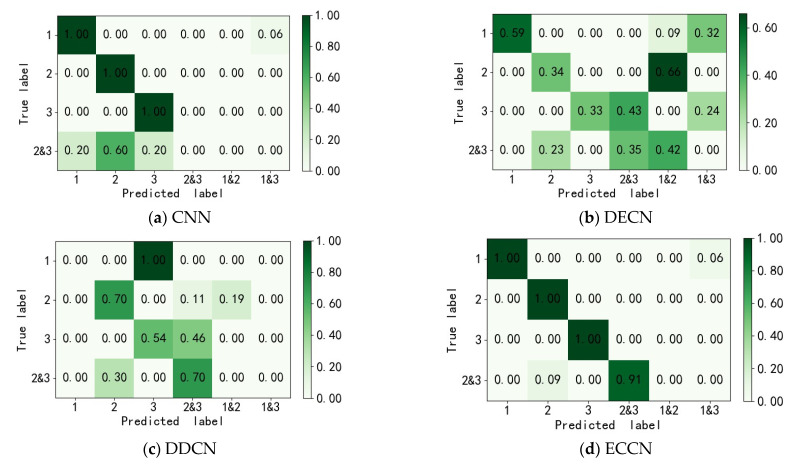
Bearing dataset classification confusion matrix (**a**) CNN; (**b**) ECCN; (**c**) DDCN; (**d**) ECCN.

The model parameters are consistent with the description in Section 3.2. The experimental results are the average of 10 experimental tests. The accuracy is shown in Table 5, and the classification effect is shown in Figure 16.

As shown in Table 5, ECCN and CNN have 100% accuracy in the three single fault states of normal bearing, inner ring fault, and outer ring fault. Compared with DECN and DDCN, the highest accuracy is 59% and 70.4%, which are increased by 41% and 29.6%, respectively. In the recognition of compound faults, ECCN has a high accuracy of 91.35%. Compared with the accuracy of 34.8% of DECN and 69.5% of DDCN, the accuracy of the compound fault recognition is increased by 56.5% and 21.85%, respectively. The experimental results show that ECCN not only has a good effect on the fault diagnosis of the bearing inner ring and outer ring. Through the learning of two types of single faults, it is also possible to identify compound faults in which the inner and outer rings fail at the same time.

In order to show the classification effect of ECCN more clearly, this paper uses the classification confusion matrix to display the classification results of the four methods. The differences in the identification of the compound faults between the different methods are compared and analyzed. As shown in Figure 16, in the CNN method, 60% of the compound fault data of the inner and outer rings are identified as outer ring faults, and 20% of the compound fault data of the inner and outer rings are identified as inner ring faults. Therefore, similar to the RV experiment, when the training data for composite faults is lacking, CNN cannot effectively identify the faults. 

Although DECN and DDCN have accuracy rates of 34.8% and 69.5% in the identification of compound faults, they have poor identification results for the three states of normal bearing, inner ring fault, and outer ring fault. Among them, 32% of the normal bearing is identified as an inner ring and normal data by the DECN method, 66% of the outer ring fault data is identified as normal, and 33% of the inner ring fault is identified as an inner and outer ring compound fault. The DDCN method divides the normal fault into an outer loop fault, 19% of the outer loop fault is identified as an outer loop plus normal, and 46% of the inner loop fault is identified as an inner and outer loop compound fault. Compared with DDCN and DECN, the proposed method not only has a better effect on bearing single fault identification, but also achieves 91% accuracy in bearing composite fault identification, and has better results in both single fault and composite faults.

## 5. Conclusions

Aiming at the problem that the traditional neural network cannot effectively identify the composite fault when the training data for the composite fault of the RV reducer is insufficient, this paper proposes a new RV reducer composite fault diagnosis method. This method combines the deep fault feature extraction ability of convolutional neural network and the powerful fault feature classification and recognition ability of capsule network. In the case of missing training data for composite faults, it is possible to diagnose composite faults only through the learning of single fault data. The experimental results show that the method can effectively identify not only the single fault of the RV reducer, but also the composite fault of the combination of the planetary gear and the sun gear. The compound fault identification accuracy rate of RV reducer is 97%, and in the real bearing inner and outer ring composite fault identification accuracy rate of 91.35%. It solves the problem that traditional convolutional neural networks cannot effectively identify composite faults without composite fault data. Compared with CNN, DDCN, and DECN, the improved ECCN has stronger fault diagnosis capabilities.

## Figures and Tables

**Table 1:** RV reducer data description.

Data	Health Conditions			
	Normal	Multitooth Wear of Planetary Gear	Multitooth Wear of Sun Gear	Compound Fault
train	1000	1000	1000	—
test	1000	1000	1000	1000
label	1	2	3	2&3

**Table 2:** ECCN Model structure parameters.

Type	Activation Function	Parameters Name	Parameters	Output Size
input layer	/	/	/	(32,768, 1)
convolution layer	leakyReLU	Kernels	150 × 32 × 2	(16,310, 32, 1)
pooling layer	/	Pooling size	2	(8155, 32, 1)
convolution layer	leakyReLU	Kernels	8 × 128 × 2	(4078, 128, 1)
pooling layer	/	Pooling size	2	(2039, 128, 1)
precapsule layer	squash	Vectors	8 × 12	(12, 8)
digital capsule layer	squash	Vectors	3 × 16	(3, 16)
output layer	/		3	(3, 1)

**Table 3:** RV reducer data classification results.

	Normal	Multitooth Wear of Planetary Gear	Multitooth Wear of Sun Gear	Compound Fault	Average Accurate Rate
CNN	100%	92%	89%	—	70.25%
DECN	94%	78%	40%	74%	71.50%
DDCN	100%	86%	93%	92%	92.75%
ECCN	**100%**	**99%**	**98%**	**97%**	**98.50%**

**Table 4:** Bearing dataset description.

Data	Health Conditions			
	Normal	Outer Race FAULT	Inner Ring Fault	Compound Fault of Inner and Outer Ring
train	200	200	200	—
test	200	200	200	200
label	1	2	3	2&3

**Table 5:** Diagnostic results of different algorithms on bearing dataset.

	Normal	Outer Race Fault	Inner Ring Fault	Compound Fault of Inner and Outer Ring	Average Accurate Rate
CNN	100%	100%	100%	0%	75%
DECN	59%	33.85%	32.7%	34.85%	40.1%
DDCN	0%	70.4%	53.6%	69.5%	48.38%
**ECCN**	**100%**	**100%**	**100%**	**91.35%**	**97.84%**

## Data Availability

The data that are presented in this study are available on request from the corresponding author.

## Nomenclature

The following main symbols are used in this manuscript:

**Symbol**	**Symbol Name**	**Meaning/Definition**
$x_{j}^{l}$	Eigenvalue	The *j-*th eigenvalue of the *l-*th convolution layer
$w_{ij}^{l}$	Weights	The likelihood that feature $x_{i}^{l-1}$ belongs to feature $x_{j}^{l}$
$b_{j}^{l}$	Biases	The magnitude of the bias measures how easy it is for the feature to generate positive/negative excitation
*	convolution	Product of eigenvalue and weight
$y_{d}^{l}$	Output features	Pooled output features
k	pooled window	Pooled window size
$O_{j}^{l}$	Fully connected layer eigenvalues	The *j-*th eigenvalue of the output of the fully connected layer.
$\hat{O_{j}}$	Softmax normalized features	$\hat{O_{j}}$ is the feature of $O_{j}^{l}$ after softmax normalization.
C	number	number of output features
$u_{i}$	Feature vector	Input feature vector.
${\hat{u}}_{j|i}$	Prediction vector	The feature vector $u_{i}$ is multiplied by the weight $w_{ij}^{l}$ to get ${\hat{u}}_{j|i}$
$c_{j|i}$	Coupling coefficients	The relationship between the input feature vector and the predicted feature vector.
$K_{l}$	Vector number	Number of predicted feature vectors
$d_{j}$	Output vector	Coupling of underlying eigenvectors
$v_{j}$	Normalized eigenvectors	$d_{j}$ Eigenvector after squash normalization
$p_{j}$	Predicted probability	The modulo length of the vector $v_{j}$
